# A midlife crisis for the mitochondrial free radical theory of aging

**DOI:** 10.1186/2046-2395-3-4

**Published:** 2014-04-01

**Authors:** Jeffrey A Stuart, Lucas A Maddalena, Max Merilovich, Ellen L Robb

**Affiliations:** 1 Department of Biological Sciences, Brock University, St. Catharines, ON L2S 3A1, Canada; 2 Mitochondrial Biology Unit, Medical Research Council, Cambridge, UK

## Abstract

Since its inception more than four decades ago, the Mitochondrial Free Radical Theory of Aging (MFRTA) has served as a touchstone for research into the biology of aging. The MFRTA suggests that oxidative damage to cellular macromolecules caused by reactive oxygen species (ROS) originating from mitochondria accumulates in cells over an animal’s lifespan and eventually leads to the dysfunction and failure that characterizes aging. A central prediction of the theory is that the ability to ameliorate or slow this process should be associated with a slowed rate of aging and thus increased lifespan. A vast pool of data bearing on this idea has now been published. ROS production, ROS neutralization and macromolecule repair have all been extensively studied in the context of longevity. We review experimental evidence from comparisons between naturally long- or short-lived animal species, from calorie restricted animals, and from genetically modified animals and weigh the strength of results supporting the MFRTA. Viewed as a whole, the data accumulated from these studies have too often failed to support the theory. Excellent, well controlled studies from the past decade in particular have isolated ROS as an experimental variable and have shown no relationship between its production or neutralization and aging or longevity. Instead, a role for mitochondrial ROS as intracellular messengers involved in the regulation of some basic cellular processes, such as proliferation, differentiation and death, has emerged. If mitochondrial ROS are involved in the aging process, it seems very likely it will be via highly specific and regulated cellular processes and not through indiscriminate oxidative damage to macromolecules.

## Introduction

The basis for the mitochondrial free radical theory of aging (MFRTA) was provided by Denham Harman [1,2], who recognized the possibility of a connection between mitochondrial oxidative phosphorylation, oxygen free radical formation, cellular damage and the general degenerative phenotype of aging. This theory continued to grow and gain acceptance and by the beginning of the next decade was suggested to be perhaps the major underlying cause of aging [3]. Originally envisioned as a collection of free radical processes that had their sources and primary targets within mitochondria, the theory had by 1981 grown to encompass non-mitochondrial targets and phenomena, including amyloid plaques in the brain and cancer. By the 21st century, the basic ideas espoused in the MFRTA had grown to include a vast array of connections between mitochondrial free radical production and age-related phenomena in most cell types, tissues and physiological processes (for example, [4-7]). Now into its fifth decade, the MFRTA has provided the basic framework for thousands of studies in the field of aging that have linked mitochondrial free radical production to cellular deficits associated with aging. As the number of publications that address the MFRTA at some level has grown, the theory has inevitably shown signs of fallibility, if not evidence of an outright midlife crisis. This situation arises in part as a result of the wealth of new information and our continually developing understanding of mitochondrial oxygen free radical metabolism, mitochondrial repair and turnover, and macromolecular repair processes elsewhere in the cells that were unavailable when the theory was first postulated. In this review, we present several key predictions arising from the MFRTA that have now been comprehensively tested and summarize these experimental results. We then briefly present a more refined view of mitochondrial ROS as participants in intracellular redox regulated processes and pathways, rather than as indiscriminately damaging toxins.

## Review

### Predictions based on the MFRTA

The modern version of the MFRTA proposes that the progenitor reactive oxygen species (ROS) superoxide (O_2_^·-^) originating from several mitochondrial enzymes, including respiratory complexes I, II and III [8] enters into a number of secondary reactions leading to other ROS that ultimately react with and indiscriminately damage cellular macromolecular structures. The affected cells accumulate such damage over time and will eventually cease to function normally, contributing to reduced physiological function, and ultimately process failure and death of the organism. The MFRTA has been an excellent theory in that it is founded on some real chemical considerations and biological observations (for example, [9]) and is readily testable.

If the MFRTA is correct, it logically follows that the ability to prevent or slow the process of oxidative damage accumulation should be associated with reduced rates of age-related tissue dysfunction and, therefore, increased lifespan. Testable hypotheses bearing on this specific idea include: (1) that the rate of mitochondrial ROS production should be reduced in longer-lived organisms, and interventions that reduce this rate should extend lifespan; (2) that the cellular capacity to neutralize ROS produced by mitochondria should be greater in longer-lived organisms and interventions that change this should affect lifespan; (3) that the capacity to prevent, repair, remove or tolerate macromolecule damage should be greater in longer-lived organisms and interventions that alter these processes should affect lifespan.

These three predictions of the MFRTA have been evaluated at length through decades of research. Although there is insufficient space here to review all of the published results, we discuss some key results and briefly summarize the work in this area. We suggest that data gleaned from inter-species comparisons, dietary manipulations and genetic manipulations have collectively failed to offer sufficient support for the MFRTA, and have thus cast significant doubt on the validity of the theory.

While the field has not succeeded in validating the original MFRTA, it has, perhaps more importantly, contributed to an evolving appreciation of the roles of ROS within animal cells extending well beyond macromolecule damage. This more comprehensive view of ROS includes their ability to participate in diverse signaling pathways that directly impact cell behaviors, such as proliferation, differentiation and death. In turn, these specific processes probably do contribute to organism aging and longevity, though in a far more nuanced way that demands considering the signaling-based effects of mitochondrial ROS on specific cellular processes. We conclude the review by highlighting the emerging roles of ROS as conveyors of information within animal cells.

### The role of oxygen in the MFRTA

**Figure 1:**
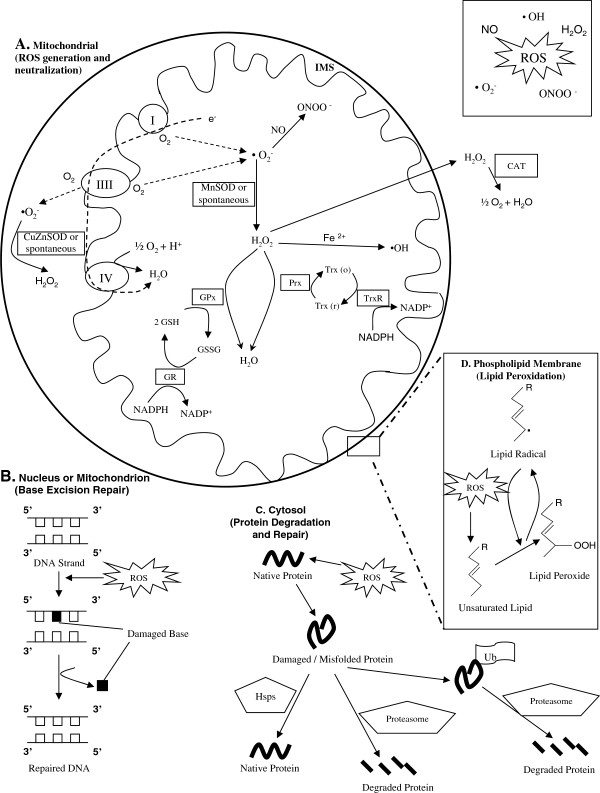
**Mitochondrial ROS generation, neutralization, macromolecular damage and repair. A**. Superoxide (O_2_^•-^) is generated in the mitochondrial matrix or inner membrane space (IMS) when an electron is donated to O_2_ (shown for complex I and III here). Superoxide produced in the IMS is converted to H_2_O_2_ by Cu/Zn superoxide dismutase (CuZnSOD). Superoxide produced in the matrix is converted to H_2_O_2_ by Mn superoxide dismustase (MnSOD). H_2_O_2_ can be neutralized to H_2_O through the action of the glutathione peroxidase (GPX)/glutathione reductase (GR) cycle at the expense of reducing equivalents (NADPH) (reduced glutathione = GSH; oxidized glutathione = GSSG). H_2_O_2_ may also be converted to H_2_O by peroxiredoxin (Prx), coupled to the oxidation of reduced thioredoxin (Trx). Oxidized Trx is reduced by thioredoxin reductase (TrxR) at the expense of reducing equivalents (nicotinamide adenine dinucleotide phosphate, NADPH). H_2_O_2_ can also diffuse into the cytosol, where it is neutralized to H_2_O by catalase (CAT) or other cytosolic enzymes (not shown). Superoxide in the matrix or IMS can form other ROS, such as peroxynitrite (ONOO-). H_2_O_2_ may also form other ROS, such as hydroxyl radicals (•OH). **B**. ROS produced by mitochondria can damage nuclear and mitochondrial DNA, causing lesions, including base modifications. These effects are countered by a variety of DNA repair processes, including the base excision repair pathway. **C**. ROS generated by mitochondria may damage cytosolic proteins. Heat shock proteins (Hsps) interact with misfolded proteins and assist in returning proteins to their native structure. Alternatively, damaged proteins can be ubiquitinated (Ub) and degraded by proteasomes. **D**. ROS generated by mitochondria can damage membrane phospholipid fatty acids via peroxidation reactions. Note that, for the purpose of clarity, this figure omits and/or simplifies some pathways involved in mitochondrial ROS metabolism*.*

O_2_ plays a major role in the MFRTA (see [10] for a review of hyperoxia and ROS), since it is one of two substrates in the reaction(s) leading to O_2_^·-^ production (the other being the electron donor, which can be a variety of molecules; see Figure 1). Turrens *et al*. [11] demonstrated the predicted linear relationship between O_2_ levels and the apparent rate of O_2_^·-^ production in submitochondrial particles. One would, therefore, predict that increased tissue O_2_ levels should be associated with increased rates of O_2_^·-^ and more rapid tissue aging, as was hypothesized by Harman [2]. Mammals have a sophisticated circulatory system with hemoglobin that shields most of their somatic cells from relatively high (approximately 21%) atmospheric O_2_, and maintains in most tissues an internal milieu closer to 3% O_2_ (see [12] for review). Therefore, it is not straightforward to vary environmental O_2_ levels and observe a concomitant effect on tissue O_2_ levels in mammalian species. However, tiny organisms like *Caenorhabditis elegans* (approximately 1 mm) that have been widely used to study the MFRTA lack both a circulatory system and hemoglobin, so O_2_ simply diffuses to the sites of its use within the animal. All *C. elegans* cells should, therefore, experience a tissue O_2_ environment that is more directly connected to that of the immediate environment. Although this species is sometimes said to inhabit hypoxic environments, it is flexible enough to flourish in normal atmosphere (21% O_2_; [13]).

Honda *et al*. [14] investigated the relationship between environmental O_2_ levels and lifespan, and found no effect when environmental O_2_ was maintained at set values between 2% and 40% over the entire lifespan. Yanase and Ishii [15] similarly found that daily exposure to 90% O_2_ did not affect lifespan in wildtype *C. elegans* and actually extended it in some strains. One explanation for the apparent lack of correlation between O_2_ and lifespan in *C. elegans* is that the organism responds by increasing its antioxidant capacity. However, in the strains in which high O_2_ extended longevity, there was no evidence of an up-regulation of any of the superoxide dismutases in response to hyperoxia exposure [15]. Similarly, genetic overexpression of these enzymes is not associated with increased lifespan [16]. A second possible explanation might be that, at higher O_2_ levels when mitochondrial ROS production might be problematic, metabolism is redirected toward glucose fermentation. However, Van Voorhies and Ward [17] showed that mitochondrial metabolism is not inhibited by O_2_ levels up to 100%, so the possible explanation that metabolic reorganization occurs to favor glucose fermentation when high environmental O_2_ levels might make oxidative phosphorylation dangerous appears also to be invalid. Therefore, higher levels of environmental O_2_, which should translate directly into higher O_2_ levels within the organism and therefore higher rates of O_2_^·-^ production in cells (if indeed antioxidant enzymes are not broadly induced), did not affect longevity in *C. elegans*.

Within some organisms (for example, humans) there are major differences in the relative exposure to O_2_ of somatic cells in different tissues. For example, some of the highest levels of O_2_ exposure in mammals occur in the lungs (approximately 10 to 14%), and one might therefore predict that lung epithelium should be particularly vulnerable to the degenerative effects of aging, especially compared to tissues like cartilage, in which chondrocytes exist in a relatively hypoxic environment (<3% O_2_). However, there is no evidence that this is so. Both Type I lung alveolar epithelial cells and articular chondrocytes have a similar mitochondrial volume density (that is, 3 to 5% [18]), suggesting similar rates of ATP turnover and O_2_ consumption, and therefore presumably also O_2_^·-^ production, yet there is no evidence that these different cell types age at different rates despite the fact that they exist in internal environments with drastically different O_2_ levels.

Within an organism, there is also a broad range of mitochondrial abundance in different cell types, ranging from 3 to 5% volume density in chondrocytes to 22 to 37% (depending upon species) in cardiomyocytes [19]. Harman [2] recognized that relative mitochondrial abundance might contribute to rates of cellular ROS production, though he considered it in the context of inter-species allometric scaling of metabolism. If ROS originating from mitochondria are responsible for aging then one would also predict that, since there should be more ROS produced within cardiomyocytes than in chondrocytes, the heart would age more rapidly (superoxide dismutase levels are similar in heart and cartilage [20]). While it is surely difficult to quantify relative rates of tissue aging within an organism, a recent epigenetic method for doing just this [21] suggests that heart tissue is actually typified by a particularly slow aging rate.

The basic differences in mitochondrial volume density (within a specific cell type) that exist among animal species are also inconsistent with a straightforward relationship between mitochondrial abundance and longevity. Some of the longest-lived endothermic vertebrate species for their respective body masses are birds and bats, even though both clades are generally characterized by relatively high mass-specific metabolic rates, and high mitochondrial abundance in heart and skeletal muscle tissues (see Robb *et al*. [22] for review).

In summary, the predicted relationships between either O_2_ and aging rate or mitochondrial abundance within cells and aging rate have not been reliably identified. It is straightforward to identify potential reasons for this lack of correlation: reduced rate of ROS production, increased ROS neutralization capacity, or superior oxidative damage repair are all possible explanations. All of these possibilities are discussed below.

### Reactive oxygen species production

Based on the above examples, it seems that the predicted simple relationships among O_2_ exposure, mitochondrial abundance and aging/longevity do not exist. One explanation for this might be that long-lived and/or high metabolic rate species have adapted to reduce the rate at which their mitochondria produce ROS. This hypothesis has been tested in many studies (see Table 1 for some examples). Sanz *et al*. [23] compared the net rates of H_2_O_2_ production in isolated mitochondria (whole flies) from three strains of *Drosophila melanogaster* with maximum lifespans ranging from 49 to 91 days, and found limited evidence for an association with lifespan. Measurements of mitochondrial H_2_O_2_ production by isolated vascular tissues of the extremely long-lived naked mole rats and Damara mole rats also failed to uncover differences compared to shorter-lived guinea pigs and mice [24]. Similarly, mitochondrial H_2_O_2_ production of isolated vascular tissue from the extremely long lived naked mole rats and Damara mole rats was found to be about the same as in shorter-lived guinea pigs and mice [24]. A similar absence of association between H_2_O_2_ generation was noted in comparisons of isolated heart mitochondria respiring on succinate (+/− the respiratory complex I inhibitor rotenone) between naked mole rats and mice [25], though in the same experiment Damara mole rat heart mitochondria did have lower H_2_O_2_ production rates than guinea pig (only in the absence of rotenone [25]). These authors also compared rates of heart mitochondrial H_2_O_2_ production in the long-lived domestic pigeon (*Columba livia*), the shorter-lived Japanese quail *Coturnix japonica* and laboratory rats. H_2_O_2_ production rates during succinate oxidation are indeed lower than in the laboratory rat, but only in the absence of rotenone. However, rates in Japanese quail were significantly higher than those in rats, despite the fact that these two species have similar maximum lifespans (MLSPs). In a similar comparison between the long-lived house sparrow *Passer domesticus* and laboratory mice, the rates of isolated liver mitochondrial H_2_O_2_ production were significantly greater in the longer-lived species [26]. When these data are expressed as the proportion of total oxygen consumed that was converted into H_2_O_2_, no between species differences are evident. Montgomery *et al*. [27] also failed to find differences in the rates of isolated liver mitochondrial H_2_O_2_ production between rats and pigeons respiring on several combinations of respiratory substrates. Indeed, these authors found that the direction of rat versus pigeon differences depended on tissue: pigeon H_2_O_2_ production rates were greater in skeletal muscle, but lower in heart muscle (respiring on succinate or succinate + rotenone). Kuzmiak *et al*. [28] also found virtually no differences in isolated skeletal muscle H_2_O_2_ production between sparrows and mice using various combinations of metabolic fuels (pyruvate, malate, glutamate and glycerol-3-phosphate). Brunet-Rossinni [29] found no consistent association between MLSP and the rates of H_2_O_2_ production in mitochondria isolated from brain, heart and kidney of the little brown bat *Myotis lucifugus* (MLSP = 34 y), the white-footed mouse *Peromyscus leucopus* (MLSP = 8 y) and the short-tailed shrew *Blarina brevicauda* (MLSP = 2 y). On the other hand, Brown *et al*. [26] showed that liver mitochondria from the little brown bat produced H_2_O_2_ at lower rates than laboratory mice when respiring on glutamate + malate. The largest and most complete single test of this hypothesis has been published by Lambert *et al*. [25] and included isolated heart mitochondria from 12 mammal and bird species. Under most experimental conditions, these investigators found few differences in H_2_O_2_ production rates between species and no association with MLSP. Only the rate of H_2_O_2_ production in mitochondria oxidizing succinate alone was correlated negatively with MLSP. Taken together, however, the collection of experimental results discussed above provides little support for the hypothesis that longer-lived organisms have adapted to produce less mitochondrial ROS (but see below for a discussion regarding the limitations of these experimental data).

**Table 1:** Hydrogen peroxide production by isolated mitochondria or tissue of relatively short- and long-lived animal species

**Species investigated**	**Tissue**	**Metabolic substrate(s) used**	Relationship to MLSP*	**Reference**
Fruit fly *Drosophila melanogaster*	Whole body	Pyruvate + proline	Weak correlation	[23]
		Glycerol-3-phosphate	Weak correlation males only	
		Glycerol-3-phosphate + rotenone		
			No correlation	
Naked mole rat *Heterocephalus glaber*, Damara mole rat *Cryptomas damarensis*, Guinea pig *Cavia porcellus*, laboratory mouse *Mus musculus*	Vascular tissue	Succinate ± rotenone	No correlation	[24]
Domestic pigeon *Columba livia*, Japanese quail *Coturnix japonica*, laboratory rat *Rattus norvegicus*	Isolated heart mitochondria	Succinate	Negative correlation	[25]
		Succinate + rotenone	No correlation	
Laboratory mouse *Mus musculus*, house sparrow *Passer domesticus*	Isolated liver mitochondria	Glutamate + malate	No correlation when standardized to total O_2_ consumption	[26]
Laboratory mouse *Mus musculus*, house sparrow *Passer domesticus*	Isolated mitochondria from pectoralis (sparrow) or hindlimb (rat) muscle	Multiple substrates	No correlation when O_2_ consumption rates considered	[28]
Pigeon (species not indicated), rat (*Rattus norvegicus*)	Isolated mitochondria from heart, muscle and liver	Pyruvate + malate	Higher rates in pigeon muscle	[27]
		Succinate		
			Higher rates in rat heart	
		Succinate + rotenone		
			Higher rates in rat heart	
Little brown bat *Myotis lucifugus*, short-tailed shrew *Blarina brevicauda*, white-footed mouse *Peromyscus leucopus*	Isolated mitochondria from brain, heart, kidney	Not given	No correlation	[29]
Little brown bat *Myotis lucifugus*, laboratory mouse *Mus musculus*	Isolated liver mitochondria	Glutamate + malate	Negative correlation	[26]
12 mammalian and avian species	Isolated heart mitochondria	Succinate	Negative correlation	[25]
		Succinate + rotenone		
			No correlation	
			No correlation	
		Pyruvate + malate ± rotentone		

*MLSP = Maximum Lifespan.

Another experimental model of reduced aging rate and increased longevity is caloric restriction, which has been used extensively to study mitochondrial ROS production. Caloric restriction often extends maximum lifespan in mice and rats, though the magnitude of the effect appears highly dependent upon strain and experimental conditions (see [30] for review). In many studies reduced rates of mitochondrial ROS production associated with caloric restriction have been reported, though there is evidence that this outcome is highly variable. Walsh *et al*. [31] compiled an exhaustive database of mitochondrial ROS production data from calorie restriction studies done with mice and rats. Perhaps surprisingly, in all tissues examined, including brain, heart, kidney, liver and skeletal muscle, the absence of effect on mitochondrial ROS production occurred almost as often as caloric restriction reduced rates of ROS production. This absence of a consistent effect is notable particularly given that positive results are more likely to be published than negative outcomes in these types of studies (for example, see [30]). We, therefore, conclude that the caloric restriction studies, as a whole, do not offer strong support for the prediction of the MFRTA that mitochondrial ROS production will be reduced.

Although the data outlined above are inconsistent with the hypothesis that a reduced aging rate is associated with reduced rates of mitochondrial ROS production, an important caveat regarding all of these data concerns how ROS production has been measured. Assumptions based on ROS measurements obtained from isolated mitochondria respiring on saturating concentrations of a single fuel in buffer equilibrated to atmospheric oxygen have limited physiological relevance. These limitations of the experimental conditions under which virtually all of our existing data have been collected have been well described (for example, see Robb *et al*. [22] for review), and are sufficiently significant that it is impossible to make strong conclusions at present. The ongoing development of *in situ* ROS probes will be important in generating more physiologically relevant data in intact cells (for example, [32]).

Another important point worth considering is that, though mitochondria may be the primary source of ROS in some cell types (particularly those with high mitochondrial abundance, though more experimental evidence is needed for this), alternate sources of ROS are clearly more important in others. For example, NADPH oxidase is a major source of ROS in activated leukocytes; peroxisomes appear to make more significant contributions to overall ROS production in liver. Brown and Borutaite [33] recently published a thoughtful criticism of what has become a dogma, that is, that mitochondria are the major source of ROS in most cells. As these authors point out, there is, in fact, only a handful of studies in which the relative contributions of various cellular sources of ROS have been quantified such that this statement can be evidence based. Even where they have been, saturating concentrations of non-physiological substrate combinations have been used and the measurements done in atmosphere-saturated buffers. Since some ROS-producing enzymes like NADPH oxidase and xanthine oxidase have relatively high K_m_(O_2_), the elevated O_2_ levels under which the measurements have been made are likely to exaggerate the contributions of these enzymes to overall ROS production. Overall, there are virtually no data that directly bears on the contribution of mitochondrial ROS production to overall rates in mammalian (or other animal species) tissues under conditions that adequately approximate physiological.

Recently, several investigators have also proposed alternative views of the role of mitochondria in the MFRTA. Brown and Borutaite [33] and Hickey *et al*. [34] suggest that, since mitochondria are capable of consuming ROS via their constituent antioxidant enzymes and cytochrome c/cytochrome c oxidase, the role of mitochondria under some physiological conditions could be as a ROS sink, rather than a source. Other investigators (for example, [35]) have suggested the hypothesis that mitochondrial ROS provides a beneficial hormetic stimulus that could enhance cellular resistance to oxidative stress by promoting the up-regulation of defense mechanisms. These interesting hypotheses, even if proven correct, would nonetheless be inconsistent with the MFRTA. Therefore, with the evidence accumulated to date using the variety of experimental approaches discussed above, the hypothesis that longevity should be associated with reductions in mitochondrial ROS production is not supported.

### Antioxidants

The second prediction arising from the MFRTA is that greater longevity should be associated with a greater capacity to neutralize mitochondrial ROS (Figure 1). Within the mitochondrial matrix Mn O_2_^·-^ dismutase (MnSOD) catalyzes the conversion of O_2_^·-^ to H_2_O_2_ in a diffusion-rate limited reaction [36,37]. The inner membrane is impermeable to O_2_^·-^ due to this molecule’s negative charge, and as the sole O_2_^·-^ dismutase in the matrix MnSOD therefore acts as a primary regulator of O_2_^·-^ concentration in this compartment and is important in controlling concentrations of ROS generated from O_2_^·-^ produced by mitochondria. O_2_^·-^ produced on the IMS side of the electron transport chain is converted to H_2_O_2_ by CuZnSOD, a primarily cytosolic antioxidant enzyme that has also been associated with the IMS. In rat liver, nearly 3% of the total cellular concentration of this enzyme is localized to the mitochondrial IMS [38]. H_2_O_2_ generated by O_2_^·-^ dismutation in the matrix may go on to be further detoxified to water within mitochondria by glutathione peroxidase (reviewed by Margis *et al*. [39]), peroxiredoxin 3 and 5 [40], and thioredoxin pathways [41] or, in heart mitochondria, catalase [42]. Mitochondrial H_2_O_2_ that is not intercepted by antioxidant enzymes in the matrix can diffuse into the cytosol, where it may be detoxified by cytosolic isozymes in the glutathione [39] and thioredoxin [43] pathways, or by the enzyme catalase [44].

Measurements of the two major O_2_^·-^ dismutases of the mitochondrial (MnSOD) and cytosolic (CuZnSOD) compartments and several enzymes involved in H_2_O_2_ neutralization (catalase and also the cycle of glutathione oxidation involving glutathione peroxidase and glutathione reductase) have been made in many of the same experimental models used for assessing mitochondrial ROS production. In a comparison of the naked mole rat and laboratory mouse, activities of MnSOD (not corrected for mitochondrial abundance) and CuZnSOD measured in liver at mid-age are significantly higher in the naked mole rat [45]. In contrast, catalase activities are not different and glutathione peroxidase activities are an order of magnitude lower in the naked mole rat liver. Page *et al*. [46] measured all five of the antioxidant enzymes listed above in brain, heart and liver tissues of 14 species of endotherm vertebrates. Of 15 tested correlations (five enzymes x three tissues), only two were positive and statistically significant. These were MnSOD and catalase in the brain, which were higher in longer-lived species, even after correction for body mass and phylogenetic effects [46]. Similar measurements of two other antioxidant enzymes, glutaredoxin and thioredoxin reductase, also failed to reveal significant positive correlations with lifespan in any of these three tissues [47]. Thus, of 21 tested associations of six antioxidant enzymes only 2 showed the hypothesized positive correlations with lifespan [46,48]. Since measurements made in whole tissue homogenates provide little insight into ROS neutralization within mitochondria, we measured glutathione peroxidase and glutathione reductase activities in brain mitochondria isolated from eight species of vertebrate endotherm (Robb *et al*. [22]). This analysis also failed to show a relationship between with MLSP, and therefore failed to support the second hypothesis relating to the MFRTA, that is, that the cellular capacity to neutralize ROS should be greater in longer-lived organisms.

Walsh *et al*. [31] recently summarized the results of several decades of studies examining antioxidant enzyme levels/activities (superoxide dismutases, catalase, glutathione metabolizing enzymes) in the context of caloric restriction. Similar to their findings with respect to mitochondrial ROS production, these authors show the absence of a consistent up-regulation of antioxidant enzymes concomitant with caloric restriction in mice and rats.

A number of mammalian lifespan studies have been conducted utilizing transgenic or knockout laboratory mouse models to increase or decrease gene expression of mitochondrial and other key intracellular antioxidant enzymes (Table 2). The results of such studies have been the in-depth focus of other review papers (see [49-51]) and, therefore, will not be reviewed in detail here. Overwhelmingly, the conclusions from these studies has been that, although the expected increases and decreases in tissue oxidative damage biomarkers are usually observed in antioxidant enzyme gene under-expressing and overexpressing individuals, respectively, there are seldom corresponding effects on longevity. Thus, the results of experiments using this approach have most often yielded results that are inconsistent with the MFRTA.

**Table 2:** Survival data for mice over-expressing or under-expressing antioxidant enzymes

**Genotype**	**Strain**	***N***^***$***^	**90th percentile survival (95% C.I.) [versus WT Control]**	**Survivorship effect? (*P *** **>0.05)**	**Reference**
***Single gene manipulations:***					
**Mn superoxide dismutase**					
***Sod2 *** **Tg**	C57BL/6 (males)	50	1,165 d (1,092 to 1,245) [versus 1,128 d (1,080 to 1,206)]	No	[49]
***Sod2 *** **Tg**	Not indicated	24	N/A - 43.0 months [versus 36.5 months] and	N/A*	[52]
***Sod2***^***+/−***^	C57BL/6 (females)	70	1,027 d (1,044 to 1,154) [versus 1,034 d (1,002 to 1,099)]	No	[53]
**CuZn superoxide dismutase**					
***Sod1 *** **Tg**	C57BL/6 (male)	44	1,121 d [versus 1,090 d]	No	[50]
***Sod1 *** **Tg (homozygous)**	CD1	119	30 months [versus 31 months]^!^	No	[54]
***Sod1 *** **Tg (hemizygous)**	CD1	200	31 months [versus 31 months]^!^	No	[54]
***Sod1***^***−/−***^	C57BL/6	10	762 d (761 to 767) [versus 1,076 d (1,035 to 1,298)]	Yes - decrease in mean lifespan	[51]
***Sod1***^***+/−***^	C57BL/6	12	N/A - 28.7 ± 1.3 months [versus 29.8 ± 2.1 months]^^^	No	[55]
***Sod1***^***−/−***^	C57BL/6	18	N/A - 20.8 ± 0.7 months [versus 29.8 ± 2.1 months^C1^]^^^	Yes	[55]
**Catalase**					
***Cat *** **Tg**	C57BL/6 (male)	44	1,099 d [versus 1,090 d]	No	[50]
***Cat *** **Tg**^**p**^	C57BL/6 ^<^	44	N/A (only a survivor curve was given - clear values not provided)	No’	[56]
***Cat *** **Tg**^**m**^	C57BL/6 ^<^	62	N/A	Yes - increased median and maximum lifespan	[56]
***Cat *** **Tg**^**n**^	C57BL/6 ^<^	78	N/A	No	[56]
**Glutathione peroxidase 4**					
***Gpx4 *** **Tg**	C57BL/6 (males)	18	1,072 d (1,062 to 1,080) [versus 1,106 d (1,026 to 1,161)]	No	[51]
***Gpx4***^***+/−***^	C57BL/6 (females)	50	1,126 ± 20 d [versus 1,145 ± 9 d]	Yes - significant increase in mean lifespan only	[57]
**Glutathione peroxidase 1**					
***Gpx1***^***−/−***^	C57BL/6 (females)	59	1,063 d (1,031 to 1,183) [versus 1,091 d (1,040 to 1,188)]	No	[58]
**Thioredoxin 1**					
***Trx1 *** **Tg**	C57BL/6 (males)^>^	41	1,134 d [versus 1,159 d]	No^#^	[59]
***Trx1 *** **Tg**	C57BL/6 (males)^>^	60	1,151 d [versus 1,143 d]	No^#^	[59]
***Trx1 *** **Tg**	C57BL/6 (females)	40	1,152 d [versus 1,230 d]	No	[59]
**Thioredoxin 2**					
**Trx2**^**+/−**^	Mix of C57BL/6 J & 129 (females)	26	1,059 d (1,020 to 1,139) [versus 1,186 d (1,086 to 1,359)]	No	[51]
**Methionine sulfoxide reductase**					
***MsrA***^***−/−***^	Mix of C57BL/6 J & 129 (males)	25	1,157 d (1,105 to 1,203) [versus 1,140 d (1,092 to 1,204)]^C2^	No	[60]
***MsrA***^***−/−***^	C57BL/6 J	17	N/A - 409 ± 33 d [versus 680 ± 71 d]^^^	Yes - increase in mean lifespan	[61]
***MsrA***^***+/−***^	C57BL/6 J	8	N/A - 672 ± 80 d [versus 680 ± 71 d]^^^	No	[61]
***Multiple gene manipulations:***					
***Sod1 *** **Tg / *Sod2 *** **Tg**	C57BL/6 (males)	54	1,075 d [versus 1,090 d]	No	[50]
**S** ***od1***^**−/− **^**/ S** ***od2***^**+/−**^	C57BL/6	11	886 d (817 to 883) [versus 1,076 d (1,035 to 1,298)]	Yes	[51]
***Sod1*** **Tg / *Cat *** **Tg**	C57BL/6 (males)	47	1,098 d [versus 1,090 d]	No	[50]
***Sod2***^**+/− **^**/ *Gpx4***^**+/−**^	C57BL/6	11	1,025 d (938 to 1,099) [versus 1,076 d (1,035 to 1,298)]	No	[51]
***Sod2***^***+/− ***^ ***/ Gpx1***^***+/−***^	C57BL/6	25	1,057 d (1,027 to 1,298) [versus 1,091 d (1,040 to 1,188)]	No	[58]
***Sod2***^***+/−***^ ***/Gpx1***^***−/−***^	C57BL/6	33	1,121 d (1,069 to 1,248) [versus 1,091 d (1,040 to 1,188)]	No	[58]
***Sod1***^***−/− ***^ ***/ Gpx1***^***−/−***^	C57BL/6 (males)	11	828 d (799 to 868) [versus 1,076 d (1,035 to 1,298)]	Yes - decrease in mean lifespan	[51]
***Sod1***^**−/− **^**/ *Gpx4***^**+/−**^	C57BL/6 (males)	16	866 d (817 to 883 [versus 1,076 d (1,035 to 1,298)]	Yes - decrease in mean and median lifespan	[51]
***Gpx1***^**-/ **^**/ *Gpx4***^**+/−**^	C57BL/6	40	1,124 d (1,086 to 1,359) [versus 1,076 d (1,035 to 1,298)]	No	[51]

Tg = transgenic overexpression.

d = days.

^$^Sample size of genetically manipulated mice groups. Sample sizes were generally similar or identical to that of WT control groups, except in [31], where *N* = 119 for control group.

N/A = not available.

*Statistical analysis was not performed. Increased maximum lifespan was observed, but no changes in mean lifespan (Means: Sod2 Tg = 28.8 months versus WT = 27.6 months).

& Max lifespan (90th percentile survival was not provided).

^!^95th percentile survival.

^^^Mean lifespan ± SEM (90th percentile survival was not provided).

^C1^Control groups consisted of Sod1^+/+^ and Sod1^+/−^ mice. Values are ± SEM.

^P^Catalase was targeted to the peroxisome.

^m^Catalase was targeted to mitochondria.

^n^Catalase was targeted to the nucleus.

^<^Two mouse lines were used.

`A slight significant extension of median lifespan was observed in one mouse line (and no extension of maximum lifespan).

^>^Two separate cohorts of mice were used in the study.

^C2^Control mice consisted of both WT and *Msr*^*+/−*^ mice.

^#^No change in mean or 90th percentile survival, but *P* <0.05 for 10th percentile survival.

One exception to this general rule has been the targeting of human catalase to mitochondria in mice, which does appear to increase both mean and maximum lifespan, although the effect on lifespan was apparently reduced when the transgenic mice were backcrossed to control for differences in genetic background [56]. Interpretation of this experimental model from the perspective of mitochondrial ROS and intracellular oxidative damage leading to aging and tissue dysfunction (reviewed in Wanagat *et al*. [62]) is complicated by the fact that human catalase expression in these mouse tissues is mosaic, with the human protein being detectable in only 10 to 50% of all cells ([56], and unpublished results from skeletal muscle). Although the authors do not provide an average number of transgene expressing cells we can assume that less than 50% either do not express the transgene or express it at very low levels that are not detectable. By extension, intracellular macromolecules within the majority of cells would presumably not have enhanced protection from mitochondrial ROS. The observed effects of the genetic manipulation on aging and age-related pathologies (Wanagat *et al*. [62]) must presumably originate from a subset of cells within the mouse tissues, and for this reason it is difficult to interpret what is happening in this experimental model strictly from the perspective of the MFRTA.

Small molecule antioxidants have been promoted extensively to the general public as anti-aging and pro-longevity supplements. The evidentiary underpinnings of this are rooted in part in the observations of pro-health effects of various plant-based foods with antioxidant constituents. Hundreds of experiments have now been completed to examine the putative anti-aging effects of vitamin E (tocopherols and tocotrienols) in a diverse range of species from protists to mammals, and the results of these experiments have been reviewed recently [63]. Vitamin E has variously been shown to have no effect, a positive effect and even a negative effect on aging/lifespan. Certainly, no clear picture of an anti-aging activity emerges in the hundreds of studies that have been conducted. This includes human studies, some of which have been terminated prematurely due to adverse outcomes (see [63] for review). A similar lack of consensus has emerged with respect to the anti-aging effects of a number of other vitamin antioxidant supplements, after many hundreds of experimental studies and clinical trials (for example, see the review by Dolora *et al*. 2012 [64]).

A variety of plant-based molecules, including polyphenolic stilbenes, such as resveratrol, have more recently been put forth as anti-aging elixirs due in part to their antioxidant activities. Although early results seemed to suggest pro-longevity properties for resveratrol, the dozens of experiments instigated by these findings failed to confirm any general positive effects. While there is some evidence for increased lifespan in *C. elegans*, it is lacking in most other species [65]. The National Institutes of Health’s Aging Intervention Testing Study (http://www.nia.nih.gov/research/dab/interventions-testing-program-itp/compounds-testing) has investigated the pro-longevity properties of a number of small molecule antioxidants, including vitamin E and resveratrol, in mice and reported no beneficial effects on lifespan.

Based on the results discussed above, the evidence for an association between small molecule antioxidant supplementation and slowed aging and/or increased longevity is insufficient to support the MFRTA. However, it is important to note that none of these tested molecules is specifically targeted to mitochondria, so the extent to which they access the organelle in any tissue or cell is likely highly variable. To address this potential limitation, some investigators have developed antioxidants conjugated to positively charged, membrane-permeant moieties that target them specifically to mitochondria. Perhaps the best studied example is the mitochondria targeted ubiquinone (MitoQ) [32]. The anti-aging properties of MitoQ have been tested in *D. melanogaster*, where it failed to extend lifespan [66]. While we await further evidence of the ability of MitoQ, or other mitochondria-targeted antioxidants, to slow the rate of aging, at this time there is no compelling evidence that reducing the rate of mitochondrial ROS production will slow aging or increase lifespan. Therefore, this line of investigation has failed to offer clear support for the MFRTA [67].

### Repair and removal of oxidative damage

In the context of the MFRTA, mitochondria generated ROS have generally been considered with respect to the damage they may cause cellular macromolecules. Cellular aging may therefore be affected by the avoidance of such damage, or by the repair or degradation of damaged cellular constituents (Figure 1). All of these predictions have been tested, and quite an extensive collection of data has accumulated over the past decade in particular. Only a brief overview of the results of these investigations is presented below. It is important to acknowledge that many of the avoidance, repair and removal activities/properties discussed below in the context of the MFRTA are also involved in processes not related to oxidative damage and so these results must be interpreted with this caveat in mind.

We tested the prediction that longer-lived organisms might have superior protein recycling or stabilization capacities, thus allowing them to more rapidly clear or refold, for example, oxidatively damaged proteins from cells. Salway *et al*. [47] measured the activity of the 20S/26S proteasome in tissues of 15 species of vertebrate endotherms ranging in MLSP from a few years to several decades and found no evidence of an association between longevity and proteasome activity. Interestingly, however, the basal levels of several heat shock proteins were found to correlate positively with longevity in the same collection of species [48]. Thus, there is some evidence that mechanisms to maintain protein homeostasis might be superior in longer-lived animal species. It is important to note, though, that this latter mechanism is not specific to oxidatively damaged proteins and, indeed, may be driven by entirely different selective pressures.

Experiments with calorie restricted rodents have produced varying results. In skeletal muscle, caloric restriction has been shown to increase [68] and to decrease [69] proteasome activity in older rats. In heart tissue of rats, Li *et al*. [70] found different results of caloric restriction on 20S and 26S proteasome activities. In liver, mild caloric restriction but not every other day feeding increased some proteasome activities, but did not affect others in aged rats [71]. Taken together, the results from comparative studies and caloric restriction are somewhat equivocal in their support for the prediction that repair and removal of oxidatively damaged proteins will be greater in longer-lived organisms. However, much more work is needed before any strong conclusions can be made.

Unsaturated phospholipids in mitochondrial and other cellular membranes are vulnerable to oxidative damage mediated by mitochondrial ROS. The hypothesis that resistance of membrane phospholipids to peroxidative damage is enhanced in longer-lived organisms has been tested by investigators over the past two decades (see [72] for review). Although there is some evidence to support this hypothesis, it is not clear whether differences in peroxidizability index (that is, the propensity of phospholipid species to undergo peroxidation reactions) are related to lifespan or to other traits (see [73]).

DNA oxidative damage is thought to be a major cause of aging (see [74] for review), with mitochondrial ROS considered to be the origin of damaging ROS in this equation. One of the major pathways for repairing oxidative damage in both mitochondrial and nuclear DNA is base excision repair (BER). (Page and Stuart [75]) measured nuclear BER enzyme activities in tissues of mammals and birds with a range of MLSPs from several years to several decades and found no evidence that they were enhanced in longer-lived species. Knockout and overexpression of BER genes in mice has similarly not often had the predicted effects on lifespan. For example, the OGG1 gene knockout mice with impaired ability to excise the common oxidative lesion 8-oxo-deoxyguanine from mitochondrial DNA are without apparent aging phenotype (Stuart *et al*. [76]). Similarly, the heterozygous knockout of polymerase β, a major BER polymerase, did not shorten maximum lifespan of mice [77].

With respect to the roles of DNA repair in longevity, it is probably important to make a distinction between the needs of post-mitotic somatic cells and those that continue to divide throughout the lifespan. For example, Page and Stuart [75] made measurements in nuclear fractions from liver and brain tissue, which is composed primarily of post-mitotic and highly oxidative cells. Park *et al*. [78] and others [73] have provided evidence that multiple DNA repair pathways, including BER, are enhanced in cultured fibroblasts established from longer-lived versus short-lived mammals. Of course, in this cell type, mitochondrial volume density is typically quite low (approximately 3%), as is the reliance of oxidative phosphorylation to meet ATP turnover needs and, therefore, mitochondrial ROS production should be moderate, particularly when the cells are grown at physiological concentrations of O_2_. In addition, as noted above, one problem with interpreting DNA repair activities strictly within the context of the MFRTA is that pathways such as BER that are involved in repairing oxidative damage also repair lesions that have no direct association with ROS.

### ROS as signaling molecules

Taken together, the results discussed above suggest that if ROS participate in the biology of aging, it is not via the straightforward processes envisioned by the MFRTA. Rather, oxidative modifications elicited by ROS appear to alter protein biochemistry by affecting specific residues within an enzyme’s active site, or within essential structural domains that participate in protein-protein or protein-DNA interactions. Oxidative modifications of specific cysteine residues are thought to be an essential component of redox signaling systems (reviewed in [79]). In all cases, the proximal environment of the oxidation-sensitive residue, including its apparent pK_a_ and its exposure to the intracellular milieu, contributes to the ease with which it is modified by ROS [80]. It is these properties that can impart specificity in the oxidative modification of proteins.

Mitochondrial ROS arise from a one-electron reduction of molecular oxygen by electron carriers and other matrix enzymes to produce the superoxide anion. This charged species is rapidly converted to H_2_O_2_ peroxide, which, unlike its progenitor superoxide, is capable of diffusing from mitochondria to the cytosol where it may subsequently alter the activities of proteins that include transcription factors and components of signaling pathways. Intracellular H_2_O_2_ concentrations are capable of fluctuating on a rapid timescale in response to internal and external cues. In addition, this particular species is relatively inert to reaction with macromolecules, a property that enables its diffusion in the cytosol and is consistent with its proposed actions as a signaling molecule [80].

ROS have been shown to participate in directing the cellular response under pathological conditions, including hypoxia, inflammatory signals, starvation and ischemia reperfusion [79,81]. In the context of animal aging, a trend towards a more oxidative environment with increasing age (for example, Cocheme *et al*., [82]) may impact the activities of a suite of signaling pathways involved in regulating lifespan and in the development of age-related disease. Beyond a function in signaling under stress conditions, a putative role for ROS in the proliferation and differentiation of animal cells has been outlined on the basis of observations made following the manipulation of ROS levels. Growth factors, such as IGF-1, VEGF and EGF, stimulate ROS production that inactivates tyrosine phosphatases, and in turn permits the propagation of signaling pathways favoring growth and division (reviewed in [83]). In contrast, overexpression of catalase or glutathione peroxidase (two enzymes that detoxify H_2_O_2_) inhibits H_2_O_2_ and serum-stimulated proliferation in endothelial cells (Ruiz-Gines *et al*. [84]; Faucher *et al*., [85]). *In vivo*, overexpression of a mitochondria-targeted catalase in mice reduces the incidence of breast cancer tumor formation in these animals, data that provide tentative support of a potential role for mitochondrial H_2_O_2_ production as a mitogenic signal *in vivo*[86]. While these data could be used to build the argument that a reduction in mitochondrial ROS production reduces cancer in older populations, it is important to note that overexpression of antioxidant enzymes that reduce intracellular ROS levels are not generally associated with increased longevity, and that the roles of mitochondrial ROS are complex.

However, the effects of H_2_O_2_ on the cell cycle are not completely straightforward, as altered intracellular H_2_O_2_ concentrations have also been reported to slow cell proliferation. For example, manipulation of endogenous mitochondrial H_2_O_2_ production via alterations in MnSOD levels has been shown to promote entry into quiescence [87], and to slow proliferation in a number of cancerous cell lines (for example, [88-90]). In human glioma cells the concomitant overexpression of MnSOD and GPx abolishes the growth inhibitory effects that are associated with MnSOD overexpression alone, suggesting that in this cell type the MnSOD-stimulated increase in H_2_O_2_ concentrations underlies changes in proliferation [91]. Thus, H_2_O_2_ may act as a signal to stimulate or inhibit cell division.

A critical aspect of ROS signaling is its ability to act in an autonomous, highly localized, largely cell-specific manner. Recently, the potential regulatory actions of ROS have been described in the maintenance and differentiation of tissue resident stem cells. Stem cells reside in low oxygen niches and are primarily glycolytic in their undifferentiated state [92]. In Drosophila, hematopoietic progenitor cells produce low basal levels of ROS, while an increase in ROS in these cells triggers differentiation into mature blood cells (Owusu-Ansah *et al*. [93]). Overexpression of H_2_O_2_ detoxifying enzymes, including catalase, impedes hematopoietic stem cell differentiation pathways and maintains stem cell populations in a quiescent state [93]. In mammalian systems, high levels of ROS in hematopoietic stem cells are associated with depletion of stem cell populations due to dysregulated p38 MAPK activity, an effect that can be corrected with antioxidant treatment [94]. Differentiation of human embryonic stem cells is accompanied by increased mitochondrial mass, increased oxygen consumption and elevated ROS concentrations [95]. An important consideration when evaluating the importance of ROS in stem cell biology is the inherent difficulty in distinguishing between ROS-specific effects and the dramatic metabolic changes that occur generally during stem cell differentiation. Within the context of the MFRTA, the ability of ROS to regulate tissue-specific regenerative capacity could have important implications in maintaining organ function and thus animal health throughout the lifespan. However, currently there is no experimental evidence with which to evaluate this idea.

The select examples outlined above, and the many others that exist within the broader literature on this topic, support a role for ROS as signaling molecules. Unfortunately, the mechanistic details of these apparent signaling functions remain vague. Further research to clarify the nature of the ROS-induced protein modifications, the identity of the affected residues and specificity of these interactions in various experimental conditions is necessary to validate the signaling function of ROS in animal cells. Similarly, it will be essential to understand the mechanisms by which ROS concentrations are regulated within the cell, and how the systems responsible for its generation and removal coordinate to support ROS signaling in complex settings. Once these ROS-affected pathways have been clearly identified, their redox-stimulated changes during aging and contribution to lifespan can be addressed.

## Conclusions

The MFRTA has stimulated an enormous amount of research into the role of mitochondrial ROS production and oxidative stress in aging and longevity. However, as it enters its fifth decade, it seems to be having something of a mid-life crisis. Virtually all attempts to control mitochondrial ROS production or neutralization have yielded unexpected and even occasionally unwanted effects on aging and lifespan. And it seems that those organisms that have (at least partly) solved the riddle of longevity have not done so by addressing the ‘ROS problem’. Thus, the MFRTA has as yet failed to offer a sufficient explanation of organismal aging as a phenomenon. Methodological limitations may be invoked to explain the inability to detect the predicted relationships among mitochondrial ROS production, neutralization, and macromolecule damage and repair in any specific context. However, it is more difficult to advance this argument in the context of the many quite different approaches that have been taken and failed to consistently validate the predictions. Whether considering the evolution of longevity by natural selection of specific traits, the extension of lifespan by caloric restriction, the ability of transgenes, gene knockouts or small molecule antioxidants to alter lifespan, the overall conclusion has been drifting toward ‘no consistent relationship between mitochondrial ROS and longevity’.

Nonetheless, investigation of the MFRTA has contributed to the increasing depth of our understanding of ROS activities in animal cells. ROS are recognized to impinge upon signaling pathways regulating all of the fundamental aspects of cell biology: the cell cycle, proliferation and differentiation, and life and death (reviewed in [96,97]). These processes must undoubtedly contribute to the aging process at some level, but the connection appears far less direct than that envisioned in the original iteration of the MFRTA. Going forward, a more nuanced view of the MFRTA that recognizes the specific properties of individual ROS, identifies the specific proteins that are redox regulated, and considers how these ROS interact with specific cell types and cellular processes may still be productive.

## Abbreviations

CuZnSOD: CuZn superoxide dismutase; GPx: glutathione peroxidase; GR: glutathione reductase; GSH: glutathione (reduced); GSSG: glutathione (oxidized); IMS: inter-membrane space; MFRTA: mitochondrial free radical theory of aging; MLSP: maximum lifespan; MnSOD: Mn superoxide dismutase; NADPH: nicotinamide adenine dinucleotide phosphate; ROS: reactive oxygen species.

## Competing interests

The authors declare that they have no competing interests.

## Authors' contributions

JAS and ELR wrote the manuscript. LAM contributed Table 1 and edited the manuscript. MM contributed Figure 1 and edited the manuscript. All authors read and approved the final manuscript.
